# The Long Way of Oxytocin from the Uterus to the Heart in 70 Years from Its Discovery

**DOI:** 10.3390/ijms24032556

**Published:** 2023-01-29

**Authors:** Claudia Camerino

**Affiliations:** 1 Department of Biomedical Sciences and Human Oncology, Section of Pharmacology, School of Medicine, University of Bari “Aldo Moro”, P.za G. Cesare 11, 70100 Bari, Italy; ccamerino@libero.it; 2 Department of Physiology and Pharmacology “V. Erspamer”, Sapienza University of Rome, P.le Aldo Moro 5, 00185 Rome, Italy

**Keywords:** oxytocin, oxytocin receptor, bone, skeletal muscle, heart, thermogenesis, obesity, Prader–Willi syndrome, Nobel Prize

## Abstract

The research program on oxytocin started in 1895, when Oliver and Schafer reported that a substance extracted from the pituitary gland elevates blood pressure when injected intravenously into dogs. Dale later reported that a neurohypophysial substance triggers uterine contraction, lactation, and antidiuresis. Purification of this pituitary gland extracts revealed that the vasopressor and antidiuretic activity could be attributed to vasopressin, while uterotonic and lactation activity could be attributed to oxytocin. In 1950, the amino-acid sequences of vasopressin and oxytocin were determined and chemically synthesized. Vasopressin (CYFQNCPRG-NH_2_) and oxytocin (CYIQNCPLG-NH_2_) differ by two amino acids and have a disulfide bridge between the cysteine residues at position one and six conserved in all vasopressin/oxytocin-type peptides. This characterization of oxytocin led to the Nobel Prize awarded in 1955 to Vincent du Vigneaud. Nevertheless, it was only 50 years later when the evidence that mice depleted of oxytocin or its receptor develop late-onset obesity and metabolic syndrome established that oxytocin regulates energy and metabolism. Oxytocin is anorexigenic and regulates the lean/fat mass composition in skeletal muscle. Oxytocin’s effect on muscle is mediated by thermogenesis via a pathway initiated in the myocardium. Oxytocin involvement in thermogenesis and muscle contraction is linked to Prader-Willi syndrome in humans, opening exciting therapeutic avenues.

## 1. Introduction

Oxytocin (Oxt) is a nonapeptide mainly produced in the supraoptic (SON) and paraventricular (PVN) nuclei of the hypothalamus. Oxt in the brain and blood has extensive functions in both mental and physical activities. Oxt appears to be involved in the regulation of the three components of body composition: bone, fat, and muscle. Oxt effects include the regulation of energy and metabolism, appetite, and effects on the gastrointestinal system (GI), skeletal, and cardiac muscle [1]. In this review, we describe the ground-breaking findings on Oxt physiology during the 70 years after the awarding of the Nobel prize for its sequencing and discovery and also our own personal journey. Special emphasis is given to the effects of Oxt on skeletal muscle and to our recent paradigm shifting hypothesis that Oxt may increase the tone of the slow-twitch muscle upregulating Oxt receptor (Oxtr) and triggering the “oxytonic contraction” after cold stress (CS) challenge in mice [2,3]. Oxt is involved in thermoregulation through a pathway in the cardiac muscle [4]. Moreover, Oxt dysregulation in brain and muscle contraction, together with a complexity of hypothalamic disorders, is directly related to the etiology of Prader–Willi syndrome (PWS) [3].

## 2. Year—1955: Oxytocin Is First Discovered for Its Effects on Uterine Contractility but Expresses Sexual Dimorphism

### 2.1. Oxytocin in Women Health

Oxt functions are mediated by Oxtrs, and, although the uterus is the organ where Oxtrs are expressed at a higher density, Oxtrs are distributed in a wide spectrum of tissues with dramatic sexual dimorphism, meaning that Oxtrs are expressed in different density to assure an effect that is specific to sex and type of tissue. Mature Oxt and the carrier neurophysin are processed from the Oxt/neurophysin 1 prepropetide [5]. It seems that the predominant role of neurophysin is to target, package, and store Oxt within secretory granules prior to release [6]. Oxt is released both locally from somatodentrites from magnocellular Oxt neurons in SON and PVN, and distally at axon terminals within the neurohypophysis that originates from magnocellular PVN and SON. Back in 1955, Oxt was first identified for its effect on uterus contractility, and this discovery was awarded with the Nobel Prize for chemistry to Vincent du Vigneaud [7,8,9]. However, the fact that Oxt was first identified in the uterus does not mean that its functions are limited to this organ, although, for the first 50 years of its life, research was limited to it. Indeed, in both sexes, Oxt generally inhibits pain perception, is anorexigenic, and augments muscle tonicity, sexual activities, and aggressiveness. However, there are significant differences in Oxt levels and distribution of Oxtrs in men from women. Thus, Oxt functions in men are different from women, particularly in reproduction. In men, the reproductive functions are relatively simple [10]. In women, the reproductive functions involve the menstrual cycle, pregnancy, parturition, lactation, and menopause. This is why Oxt regulation of women’s health and disease is a unique topic of physiological and pathological studies. Indeed, women’s biological activities are regulated not only by the hypothalamic–pituitary–gonad (HPG) axis but also by Oxt. While Oxt commonly influences sexual behavior, production of sex steroids, and the maturation of gemmates of both sexes, it differently influences women’s health and disease at different reproductive stages because Oxt and Oxtrs are differently expressed in various organs, and these histological features allow Oxt to modulate body functions differently and with different patterns including neuromodulation, neurosecretion, endocrine, autocrine, and paracrine effects. Under the regulation of these extracellular and intracellular factors, Oxt neuronal activity and Oxt secretion can meet the demands of body activities in response to environmental changes. In mammals, Oxtrs have been identified in a broad spectrum of tissues, including the kidney, heart, thymus, pancreas, adipocytes, and other sites in addition to the central nervous system (CNS) [6,10]. Expression of Oxtrs in the hypothalamus, uterus, and mammary gland is stimulated by estrogens [11]. In females, Oxtrs are specifically localized in myoepithelial cells of the mammary glands and in the myometrium and endometrium of the uterus. Peripheral actions of Oxt are commonly associated with smooth muscle contraction, particularly within the female and male reproductive tracts [12]. In the brain and spinal cord, activation of Oxtrs is associated with a variety of mental activities such as social memory, pair bonding, maternal behavior, and aggression, as well as instinctive behaviors such as sexual activity, anxiety, feeding, and pain perception, all related to reproduction. By innervating median eminence and median preoptic area, Oxt can increase gonadotropin-releasing hormone (GnRH) release and the activity of the HPG axis [10,13]. By contrast, circulating and locally produced Oxt can influence body functions at cellular, tissue, and organ levels.

### 2.2. Oxytocin in Men and Women

Oxt extensively modulates body functions, although significant differences between males and females emerge. Sexual dimorphism of Oxt functions is based on expression levels of Oxt and Oxtrs. For instance, serum Oxt is significantly higher among women than men [14], making women more sensitive to Oxt reduction, which accounts for menstrual pain [14]. By contrast, Oxt binding sites in the ventromedial hypothalamus (VMH) and dorsal horns are significantly higher in males vs. females [10], which may contribute to the central regulatory actions of Oxt on feeding. Males also exhibit higher Oxtr levels in the medial amygdale irrespective of the reproductive status [15], which likely makes men less fearful facing stressful challenges because Oxt acting on the medial amygdale inhibits fear. Higher Oxtr levels in the nucleus accumbens are present at breeding males but not females [15], which makes paternal behaviors conditional [16] and more rewarding [10,17,18]. This sex dimorphism in the distribution of Oxt and Oxtrs sets a histological basis for gender specific functions and behavior. Oxt can extensively modulate body activities at peripheral sites and is a pivotal regulator of male reproductive functions, although, among all Oxt functions, the most dramatic sexual dimorphism is in reproduction. Oxt increases during sex arousal in males and females and in the CNS. Oxt from the PVN in the ventral tegmental area (VTA) initiates a pathway that involves activation of dopamine, glutamate, and other neurons in the VTA, triggering the motivational and rewarding aspects of sexual behavior [19]. Oxt release from Oxt neurons does not increase during pregnancy until the time shortly before parturition. Thus, the development of Oxt/Oxtr signaling is an adaptive response for maintaining the safety of pregnancy. However, increased Oxt synthesis and preterm Oxt release in the hypothalamus are necessary for the maturation of hypothalamic machinery that allows Oxt to be released in intermittent pulses during parturition and lactation [10]. Thus, Oxt actions during pregnancy highly match peripartum physiological demands. Plasma Oxt and nocturnal uterine activity increase progressively during late pregnancy and delivery [10]. This is associated with the effect of light/darkness on the pulsatile Oxt release [10], which determine the high incidence of parturition during the night. Regarding the lactation-associated health issue, it has been extensively accepted that normal breastfeeding can reduce the incidence of postpartum depression, maternal obesity, and diabetes. Oxt is necessary for these benefits of breastfeeding. Moreover, cardiovascular diseases increase dramatically in postmenopausal women. When estrogen production decreases, the activation of estrogen receptors on pre-autonomic PVN Oxt neurons is also weakened. As a result, Oxt regulation of the HPG axis is weakened. By contrast Oxt can protect the cardiovascular system by maintaining cardiovascular integrity, suppressing atherosclerotic alterations and coronary artery disease, and promoting regeneration and repair injuries [20]. Thus, Oxtr/Oxt protection is suppressed more strongly in the ischemic myocardium in females than males. It also accounts for why females have a higher risk of heart failure and death following myocardial infarction relative to males [21]. These results show that Oxt has common effects in both male and female physiology, but show dramatic differences in reproductive regulation.

## 3. Year—2007: Oxytocin Is in Bone

### 3.1. In Vitro Studies

In early 2007, the anabolic effect of peripheral Oxt on bone was demonstrated. Bone cells express Oxtrs, and Oxt promotes osteoblast (OB) differentiation and function, leading to an increased bone formation with no effect on bone resorption and an improvement of bone microarchitecture. Oxt is synthesized by OB, and this synthesis is stimulated by estrogens. Animal studies demonstrate a direct action of Oxt on bone, as the systemic administration of Oxt prevents and reverses the bone loss induced by estrogen deficiency. Although Oxt is involved in bone formation in both sexes during development, Oxt treatment has no effect on male osteoporosis [18]. Bone mass is maintained by the balance between bone formation by OB and bone resorption by osteoclasts (OC). Oxt negatively modulates adipogenesis [22]. The anabolic action of Oxt appears to be related in part to a direct effect on its receptor expressed on OB [18]. Estrogens are known to stimulate Oxt synthesis in bone as in other tissues [6,23]. Estrogens positively stimulate Oxt production by OB, through activation of the MAP kinase/ERK pathway and Oxtr expression by a genomic mechanism of action [18]. These two effects are synergistic through a local feedforward loop, as there is an autocrine/paracrine secretion of Oxt by OB induced by estrogens [18]. Although Oxt stimulates OC differentiation, it also inhibits the activity of mature OCs, resulting in no effects on bone resorption because Oxt reduces osteoprotegerin expression and increases RANKL expression by OB promoting OC differentiation [18]. However, in an in vitro culture of OB where the precursors were treated with Oxt, the number of OCs was increased but their ability to resorb was diminished. The decreased resorbing capacity of OC induced by Oxt is explained by the ability of Oxt to increase intracellular calcium that increases NO production, diminishing OC activities [18]. Unexpectedly, Oxt/Oxtr knockout mice develop high bone mass secondary to obesity and low sympathetic tone [24,25].

### 3.2. Ex Vivo Studies

In ovariectomized (OVX) rats, intraperitoneal injection of Oxt prevents the decrease in the number of OBs and osteocytes, as well as in the osteoprotegerin/RANKL serum ratio, and the increase in bone turnover markers [18]. Oxt has a direct action on the skeleton that appears to be related to a peripheral action of Oxt and not to an indirect action through the CNS [24]. At the tissue level, the Oxt treatment improves the microarchitecture. The beneficial effects on bone density and microarchitecture of Oxt systemic administration have been confirmed by other studies in OVX rats and rabbits [26]. Indeed, the systemic administration of Oxt promotes peri-implant bone healing and osseointegration of titanium implants [27]. Marrow fat content increases with trabecular microarchitecture deterioration and is connected to the prevalence of bone fracture in osteoporosis [28,29]. In Ovx mice, a subcutaneous injection of Oxt reverses bone loss assessed using micro-computed tomography and reduces bone marrow adiposity by decreasing marrow adipocyte density [22]. Oxt serum levels were not correlated to any other measured neuro-pituitary hormone, including leptin and estradiol, and logistic regression analysis showed that osteoporosis status remained significantly correlated to Oxt serum levels regardless of age [18]. These data reinforce the fact that the anabolic effect of Oxt on bone is related to a direct and peripheral action on bone cells independently of estradiol–Oxt-mediated action [30,31]. In line with animal studies regarding the sex-specific action of Oxt in a large prospective cohort of men (MINOS), Oxt serum levels were not associated with BMD bone turnover rate or prevalent fractures [30,31], but serum Oxt level was significantly lower in men with severe osteoporosis compared to men with normal bone status suggesting the effects of Oxt on other determinants of fracture risk such as muscle strength [30,31]. As Oxt has pleiotropic effects, the therapeutic perspective is very promising. Indeed, Oxt has wide implications for general health; Oxt is a stress-coping molecule with anti-inflammatory and antioxidant properties, influences the immune system, body composition, cognitive functions, and mood, and has been tested in the treatment of numerous diseases including anxiety, pain, diabetes, cardiovascular diseases, and breast cancer [32,33,34]. Oxt requires a proper transport system to be delivered to the desired cells and tissues, thereby enabling the activation of the Oxtr in the target cells. In this regard, nanomedicine and the development of delivery systems represent a very active research area including the administration of nanoparticles carrying different compounds, including Oxt. However, there are currently no human data on the beneficial effects of Oxt as a treatment of postmenopausal osteoporosis.

## 4. Year—2009: Oxytocin Is in Fat and Is Involved in the Onset of Metabolic Syndrome

More than 10 years ago, our laboratory raised the notion that the hormone/neurotransmitter Oxt is related to the regulation of energy and metabolism. It all started when we noticed that mice homozygous for deletions of Oxt/Oxtr develop late-onset obesity and metabolic syndrome. Oxt and Oxtr knockout mice develop high bone mass secondary to obesity and low sympathetic tone [24,25]. What sparked our interest at that time was that Oxt- and Oxtr-deficient mice developed their metabolic phenotype in the absence of hyperphagia. This is in contrast to the expectation that hypothalamic Oxt decreases food intake by increasing leptin concentration in plasma [35,36,37]. Moreover, the metabolic role of Oxt is different in young versus older animals or it takes time to reach full force. This concept was named in our laboratory “the oxytocin paradox”. Several explanations have been given to this discrepancy, including that Oxt may only mark the identity of neurons projecting from PVN, but its action is mediated by classical neurotransmitters such as GABA; alternatively, Oxt may be anorexigenic in normal mice, but developmental mechanisms may compensate for its absence in Oxt^−/−^ or Oxtr^−/−^ mice [3,36,38]. The appetite of Oxt^−/−^ reported as normal, in spite of the hyperleptinemia, was possibly excessive relative to the level of adiposity [39]. This was not the case since the stomachs of Oxt-deficient mice were reported comparable to wildtype mice for size and weight, ruling out any excess in food consumption [36,40]. The tipping point of these observations was that Oxtr-deficient mice are thermogenically impaired, with a basal temperature lower than wildtype. This shed a light on the role of Oxt on temperature regulation and lean/fat mass composition of this model [35]. However, the lean/fat mass composition specific to skeletal muscle could be the reason for the normophagic obesity in this model. From this first point, it took us about 10 additional years of study to come to the conclusion that the effects of Oxt on metabolism and energy are both direct, as Oxt is anorexigenic, and indirect, as Oxt acts specifically on muscles potentiating the majority of the slow-twitch muscles, as well as the uterus [3,35]. The normophagic obesity of Oxt^−/−^ mice was probably caused by a general muscular loss of function that slowly increased the intramuscular adipose tissue and ectopic fat accumulation in skeletal muscle and ultimately drove the late-onset obesity and metabolic phenotype rather than increased food consumption. The presence of concomitant sarcopenia and obesity confers worse functional outcome compared to either alone. Nevertheless, the study of Oxt in skeletal muscle and fat accumulation needs further investigation. Studies on genetic models of obesity have highlighted that nutritional status does not always determine Oxt concentrations in blood. For example, in ob/ob mice, which are homozygous for leptin expression, no difference in serum Oxt was detected relative to wildtype, whereas, in db/db mice, which are leptin-resistant because they lack the long isoform of the leptin receptor Ob-Rb, serum Oxt concentrations were decreased relative to lean control mice [12,41]. Of note, it was also interesting that exogenous administration of Oxt improves sarcopenia and muscle mass [12,42]. This indeed represented the initial evidence that Oxt regulates body composition through thermoregulation.

## 5. Year—2016: Oxytocin Regulates Thermogenesis and “The Oxytonic Effect”

### 5.1. Oxytocin in Muscle Adaptation after Cold Stress Challenge

Oxt regulates a diversity of social behaviors related to reproduction. Indeed, Oxt concentration increases during challenging situations including pregnancy and lactation, triggering aggressive behavior that is important after labor for the protection of the offspring when the offspring is most vulnerable to predators and Oxt concentration in plasma is at its peak [43]. Consistent with this knowledge, we hypothesized that Oxt may increase muscle tone to ensure a better response to the “fight response”. Hence, to trigger skeletal muscle contractions activated by Oxt, we elaborated a model of CS exposing mice to 4 °C for a shorter or longer time [2,44,45]. The thermogenic challenge increases Oxtr mRNA expression in Soleus muscle (Sol) and decreases circulating Oxt following a negative feedback loop in brain. The increase in Oxtr mRNA in skeletal muscle is phenotype-dependent, with Oxt potentiating the slow-twitch muscle phenotype through the regulation of myosin heavy chain 1 (slow oxidative)/myosin heavy chain 2b (fast glycolytic) ratio after CS, consistent with the shivering needs of thermogenesis. Oxt mRNA increases in bone after CS to balance the decreases in circulating Oxt. We concluded that Oxt increases skeletal muscle tonicity in the same manner it does with the uterus, triggering what we called “the oxytonic contractions” after CS. Specifically, we explored the involvement of Oxtr/TRPV1 genes and Oxt on the adaptation of skeletal muscle to CS in mice. Oxt/Oxtr mRNA was measured in Sol and Tibialis anterioris (TA) by RT-PCR. Oxtr expression was analyzed in PVN and SON and hippocampus (HIPP) by immunohistochemistry, and circulating Oxt was measured in plasma. Potentiation of slow-twitch muscle after CS is observed in rat and mice [44,46]. Oxt may lead to the activation of transmembrane ion channels permeable to calcium ions such as the TRPV1 cation channel, which plays a key role as a thermal and analgesic effector in different tissues [47]. TRPV1 mediates the pain signaling of Oxt in neurons and Oxt may directly interact with TRPV1 as previously seen for Oxt analogues in invertebrates [48,49,50]. Oxt/Oxtrs are implicated in the regulation of energy homeostasis [35,36]. Oxt/Oxtr^−/−^ mice show late-onset obesity but are normophagic, and this is probably caused by reduced metabolic rate and energy expenditure [35,36]. Oxtr^−/−^ mice are thermogenically impaired and show decreased core body temperature after acute exposure to cold [35,51,52]. Skeletal muscle is also a source of heat in CS animals and humans through voluntary contractions from exercising muscle or involuntary as contractions from shivering muscle [53]. CS activates the involuntary activation of skeletal muscle movements [54]. Oxtr is present in human myoblasts [55,56]. Oxt was first described for its tonic smooth muscle regulation of gastric motility, showing that exogenous Oxt excited circular muscle strips and isolated smooth muscle of the gastric body and contracted the slow-twitch muscle of mammary gland and myometrium [57,58]. On the basis of this rationale in an interorgan approach to the physiology of CS, we formulated the hypothesis that Oxt may contract all the slow-twitch muscles as Oxt contracts the uterus, having a tonic, thermogenic, and analgesic effect [3], and that the metabolic syndrome of Oxt/Oxtr^−/−^ mice was caused by muscular failure and depotentiation rather than increased food consumption. However, the main peripheral effects of Oxt are located in adipose tissue rather than skeletal muscle, as the expression levels in white adipose tissue (WAT) were comparable to classical Oxt target tissues [59]. Nevertheless, the expression level of Oxtr in skeletal muscle increases after thermogenic stress and is phenotype-dependent [3,60], as shown by the increase in myosin heavy chain 1 (slow-oxidative)/myosin heavy chain 2b (fast-glycolytic) (Mhc1/Mhc2b) gene expression ratio in Sol but not in TA muscle, together with the upregulation of the Oxtr gene in Sol muscle [2]. Brain Oxt may upregulate the short-term response of Sol, while it may downregulate the brain–Sol intercommunication after long-term exposure to CS, as shown by a linear correlation curve in a feedforward/feedback regulation between brain and Sol [2,44]. This means that low circulating Oxt levels are required for a better response to long-term CS challenge. Nevertheless, the Oxt signaling is maintained by the upregulation of Oxtr gene found in Sol muscle after long-term CS that balances the low level of circulating Oxt, consistent with previous studies [61]. In vivo data confirmed the in vitro data since Oxt expression in hypothalamus and Oxtr expression in adipose tissue were induced by CS, regulating both shivering and non-shivering thermogenesis [3]. Oxtr expression in PVN and Hipp increased after both long- and short-term CS exposure, as shown by immunohistochemistry, consistent with gene expression data in whole brain [12,45]. A different pattern of Oxtrs was observed in SON, the major site of Oxt secretion where Oxt was unchanged at 6 h and decreased at 5 days. The circulating levels of Oxt were unaffected after 6 h, but decreased after 5 days, consistent with in vitro data [2].

### 5.2. The Oxytonic Effect

The pathway described above was named in our laboratory “the oxytonic effect”. The actions of Oxt can be mediated by Oxtr that is a type A GPCR responsible for the release of calcium from the intracellular stores and activation of PKC. The TRPV1 cation channel is a thermal and analgesic effector in different tissues. TRPV1 mediates the pain signaling of Oxt in neurons. Circulating Oxt, in addition to Oxtrs, can directly interact with TRPV1 [47,50]. This is consistent with the hypothesis that Oxt has analgesic effects. CS induces the expression of TRPV1 and Oxtrs in skeletal muscle and is higher in slow-twitch skeletal muscle. Circulating Oxt leads to activation of Oxtrs and TRPV1 channels on the membrane. Oxtr and TRPV1 genes increased after 6 h and 5 days CS in Sol and TA. Regression analysis showed a significant linear correlation between Oxtr and TRPV1 in Sol and to a lesser extent in TA. The correlation between Oxtrs and TRPV1 in Sol and TA was lost at thermoneutrality, consistent with the coupling between these two genes at CS [3]. However, recent data have also shown that direct Oxtr stimulation inhibited lysosomal and proteolysis in rat oxidative skeletal muscle associated with Akt/FoxO1 pathway activation. Muscle incubation with an Oxtr-selective agonist did not alter protein synthesis, but in vivo short-term Oxt treatment intensified this process that resulted in Sol muscle mass gain, indicating that Oxt in vivo effects may be indirect through mediators not yet determined [62]. These in vivo Oxt effects in muscle anabolism could be mediated by the stimulation of the sympathetic autonomic nervous system, since there is evidence that Oxt stimulates secretion of adrenaline and sympathetic preganglionic neurons [63]. Oxt KO mice have less adrenaline release and develop sarcopenia [36,42]. From this perspective, it is known that the sympathetic nervous system directly innervates skeletal muscle fiber [64], inducing an anabolic effect in the skeletal muscle protein metabolism. It is possible that the in vivo Oxt treatment induces greater sympathetic activity, which can drive increased protein synthesis and muscle hypertrophy and vasodilatation [62].

The hearts of CS mice were also examined. Cardiac muscle is an Oxt target organ, expressing Oxtrs; Oxt is cardioprotective and prevents fibrosis, hypertrophy, and inflammation [65,66]. Oxt treatment prevents cardiomyopathy of db/db independently of hyperphagia and hyperleptinemia caused by a direct effect on cardiac muscle. We speculated that Oxt protects the cardiac muscle from necrotic process and increases its tonicity, as shown by histological studies and by the fact that mice rescued their body weight after CS treatment, which is a sign of good health [3]. This represented the first evidence that the thermogenic pathway initiated by Oxt involves the myocardium [3,4].

## 6. Year—2022: Oxytocin Is in the Heart

### 6.1. Beneficial Effect of Oxytocin in Coronary Artery Disease and Atherosclerosis

Oxt/Oxtrs are present in rodent and human heart [67], indicating an autocrine and paracrine role for this peptide in the myocardium. Oxt is involved in differentiating stem cells into cardiac lineages and stimulates differentiation of endothelial and smooth muscle cells, promoting angiogenesis [68]. These findings suggested that Oxt serves as a cardiomorphogen. The beneficial effects of Oxt on infarct size and functional recovery of the ischemic reperfused heart are well documented, and Oxt augments cardiomyocyte viability and function by activation of Pi3K and AKT phosphorylation and signaling [69]. Indeed, systemic administration of Oxt has significant effects on vascular tone and pressure and cardiovascular function [69], consistent with Oxt’s beneficial effect on muscle [12,61] but is impaired in an obese model deficient in Oxtr [35]. Oxt regulates arterial blood pressure through central and peripheral mechanisms via the sympathetic system [69]. The major actions of Oxt include regulation of chronotropy and inotropy of the heart, as well as vascular tone and cardiac resistance vessels. Oxt is also involved in blood pressure and body volume regulation via the cardiac and renal axis, as well as the release of atrial natriuretic peptide and NO. In addition to cardiovascular regulation and protection, Oxt exerts antioxidative and anti-inflammatory effects in cardiomyocytes. Animal studies indicate that Oxt is not only a cardiovascular-protective peptide but also important in reducing the severity of cardiovascular pathologies [69] such as atherosclerosis in coronary artery disease (CAD) [70].

### 6.2. Oxytocin in the Myocardium during Thermogenesis

**Figure 1:**
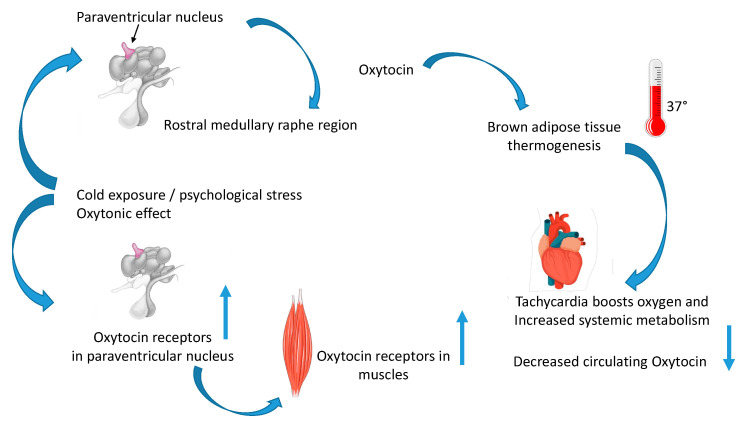
Oxytocin increases thermogenesis in muscles and heart. After cold stress, oxytocin activates the paraventricular nucleus (PVN)–rostral medullary raphe region (mMR) pathway through activation of oxytocin receptors in the mMR, evoking brown adipose tissue thermogenesis and tachycardia, and potentiating the sympathetic response via glutamatergic transmission in mMR in mice. The PVN–mMR–oxytocin pathway links the hypothalamic circuit for energy homeostasis to thermogenic and cardiac sympathetic outflow [4]. After cold stress, oxytocin receptor mRNA increases in PVN and circulating oxytocin decreases. Oxytocin receptor mRNA is also higher in the slow-twitch muscle of cold-stressed mice, leading to increased tonicity of the slow-twitch muscles. This pathway was called the “oxytonic effect”, and its defects may cause obesity and Prader–Willi syndrome [3].

Interestingly, the cardiac muscle seems to be the merging point of the normophagic obesity in our model of Oxt deficiency [35,36] and thermogenesis [12,61,71]. Indeed, the central neural pathway that regulates brown adipose tissue (BAT) thermogenesis involves the preoptic area, dorsomedial hypothalamous, and rostral medullary raphe region (rMR) [4]. The rMR harbors vesicular glutamate transporter 3-expressing sympathetic premotor neurons that innervate BAT to sympathetic preganglionic neurons [4]. These premotor neurons are activated by thermogenic signaling elicited by cold exposure, pyrogenic stimulus, and psychological stress [4]. Oxt activates a PVH–rMR–Oxt pathway that evokes BAT thermogenesis and a cardiac response that increases metabolic rate and glucose metabolism. Specifically, the development of a genetic tools made it possible to investigate the physiological mechanism via which hypothalamo-medullary Oxt innervation affects the premotor controls of BAT thermogenesis, metabolism, and cardiovascular functions [4]. Because medullary nuclei, including the rMR, regulate metabolic activity of the whole body through the autonomic nervous system, the medulla oblongata is a promising site of the metabolic action of Oxt. The central circuit mechanism via which hypothalamic Oxt neurons exert the thermogenic and metabolic effects has yet to be determined. However, Oxt neurons in the PVN are functionally related to thermoregulatory sympathetic premotor neurons in the rMR. Oxt released from PVN in the mMR stimulates BAT thermogenesis and cardiac function triggering tachycardia and leading to an increase in systemic metabolism. Oxt affects the rMR not only as a modulator that potentiates glutamatergic excitation of the thermogenic sympathetic premotor neurons but also as a transmitter that drives BAT thermogenic and cardiac responses even without glutamatergic inputs. Elevation of heart rate and tachycardia accompanying BAT thermogenesis increases cardiac output and boosts oxygen and nutrient supplies to BAT, required for its thermogenic function, and it facilitates systemic distribution of heat produced in BAT, consistent with the enhancement of body functions and mental capacity at the face of an emergency, in line with the physiological role of Oxt in the management of challenging and stressful situations and aggressiveness [43]. In this way, Oxt induces and potentiates BAT thermogenesis, increasing systemic energy expenditure (Figure 1), and chronic defects in Oxt signaling result in the development of obesity [36] and are involved in Prader–Willi syndrome (PWS) [3,61,72], as explained in the next section.

## 7. Year—2025: The Therapeutic Promise of Oxytocin and Prader–Willi Syndrome

### 7.1. Oxytocin as an Anti-Obesity Medication

Moreover, Oxt is a promising therapeutic agent to treat obesity. The effects of Oxt also differ in obese and diabetic state. Intranasal administration of Oxt in human subjects was associated with weight loss and improvements in insulin sensitivity, pancreatic function, and lipid homeostasis, strongly suggesting a role for this system as a therapeutic target in obesity and diabetes. The complexity of obesity and the pathogenesis of obesity-related metabolic complications underscore the need to better understand the role of Oxt in metabolic functions [59]. Oxt seems to affect energy regulation acting on specific areas of the brain as the hindbrain and activating the firing of catecholamine neurons in the nucleus tractus solitarius of rats [73]. Moreover, in a study on men with overweight and obesity, intranasal Oxt reduced the synchrony in activation between neural systems in a context-dependent manner, measured by functional magnetic resonance imaging. In this study, Oxt attenuated the functional connectivity between ventral tegmental area and food motivation brain regions in response to high-calorie food images, probably because Oxt prioritizes the motivation for mate searching and reproduction, in addition to food craving [74,75,76]. Recently, as proof of concept to this theory, a randomized, double-blind, placebo-controlled clinical trial was designed to study the effect of intranasal Oxt on body weight loss on nondiabetic adults with obesity [77]. Oxt was administered four times daily over 8 weeks, and body weight and vital parameters were assessed during the study and 6 weeks after treatment completion. The study reported that the patients treated with Oxt showed a significant BMI reduction after 8 week treatment with 24 international units, four times a day, of intranasal Oxt compared to placebo [77]. Nevertheless, the pharmacological use of Oxt to treat obesity has many limitations, which is why two Oxt synthetic analogues, ASK1476 [78] and ASK2131 [79], longer-lasting and with improved pharmacokinetics, were recently tested in diet-induced obese (DIO) rats for a 22 day period [79]. Both peptides reduce body weight by decreasing food intake, although this study does not include effects on energy expenditure in vivo that need to be addressed, as well as the effects on both genders. Both ASK1476 and ASK2131 showed superior efficacy as a selective Oxt agonist and increased half-life. Neither molecule is active at the vasopressin receptor V1aR, while both peptides are still potent activators of the vasopressin receptor V2R, although ASK2131 does not cause any change in water intake. Nevertheless, ASK1476 presents several side-effects such as mild toxicity at high doses and malaise. Both peptides are administered via subcutaneous injections, and their effects on food intake probably happen through the activation of vagal afferent neurons, since Oxt presents a reduced ability to cross the blood–brain barrier (BBB) from the periphery [79,80]. However, while ASK1476 was designed to be non-brain-penetrating. it is unknown if ASK2131 crosses the BBB. ASK2131 achieved a greater anorectic effect with 55% reduction in caloric intake at lower doses compared to the 34% reduction in ASK1476 achieved at significantly higher doses. Both peptides achieve a reduction in food intake effects at doses significantly lower than the primitive peptide Oxt [79]. The anorexigenic hormone leptin and Oxt present similarities that are important for the regulation of food intake and body weight. Treating high-fat diet-fed hyperleptinemic mice with Oxt re-established a normal anorexigenic effect and body weight after acute but not chronic treatment with leptin, since Oxt and leptin act synergistically to activate the CNS centers involved in the control of food intake. Oxt-induced body weight loss is mainly food intake-independent, as chronic Oxt treatment also decreased body weight without long-term modification of food intake [80].

### 7.2. Oxytocin in Prader–Willi Syndrome

Oxt dysregulation is involved in PWS, which is a rare neurodevelopmental disorder due to the absent expression of paternally active genes in the chromosome 15q11.2-q13 region. PWS patients are hypotonic at birth and have feeding problems as infants, physical and cognitive developmental delay, hyperphagia and obesity at childhood, behavioral problems, hypogonadism, and short stature, suggesting abnormalities of the hypothalamic–pituitary axis. When PWS patients are treated with recombinant growth hormone (rhGH), improvement is seen with increased muscle size and decreased fat mass [81,82]. In two different clinical trials, intranasal Oxt was administered in a double-blind, placebo-controlled, crossover study to PWS patients. These patients seem to benefit from the administration of Oxt in appetite drive, socialization, anxiety, and repetitive behavior, although a more extensive study is needed with a special focus on safety and the ability of Oxt to cross the BBB [83,84]. This is why we hypothesized that PWS patients have a defect in Oxt transmission [3,85]. Indeed, PWS are hypotonic at birth, and this hypotonic state proceeds in late-onset obesity. Postmortem assays showed that PWS males showed reduced Oxtr gene expression and density in PVN [86]. To evaluate Oxt biology in PWS, overnight fasting plasma Oxt levels in children with PWS compared to healthy unrelated siblings without PWS were examined [87]. Plasmatic Oxt levels were higher in PWS patients compared with unaffected siblings, and the diagnosis of PWS predicted Oxt levels. The symptoms of hyperphagia and behavior seen in PWS may be related to the disruption of Oxt responsiveness or feedback in PVN. This phenotype of PWS patients is the striking mirror image of the phenotype of cold-stressed mice in our model of thermogenic stress [3], where Oxtr mRNA increased in PVN, whereas Oxt decreased in plasma after CS in mice. Oxtr mRNA was also higher in slow-twitch muscle of cold-stressed mice. This led to increased tonicity of the slow-twitch muscles [3]. Since an important hallmark of PWS is, in fact, hypotonicity at birth [85,88], we hypothesized that this hypotonic state led to late-onset obesity at older age. Nevertheless, this hypothesis needs to be validated by an extensive study of Oxt/Oxtrs in different skeletal muscles of PWS patients, which is missing. In sum, a reduced Oxtr expression in PVN leads to increased Oxt secretion by the posterior pituitary due to the loss of negative feedback. The upregulation of Oxtr in PVN and in Sol muscle after CS balances the decrease in circulating Oxt. Oxt has a beneficial “oxytonic effect” in skeletal muscle through the adaptation of Oxtr/TRPV1 after long-term CS with analgesic effects and increasing its tonicity in our model of cold stress [3]. This “oxytonic effect” may be lacking in PWS patients, and the significance of Oxt in PWS patients may be an important step in developing new treatments. PWS has high circulating Oxt. This is in contrast to the expectation that hypothalamic Oxt decreases food intake by increasing leptin concentration in plasma. This may be caused by a sort of “Oxt resistance” as previously seen for leptin [89] or for high levels of inactive forms and not enough of the active forms. Alternatively, Oxtr may be suppressed after genetic or methylation defect (Figure 1). This novel pathway that we described in skeletal muscle [3] is the first evidence that Oxt involvement in thermogenesis is related to PWS.

## 8. Strengths and Limitations of Oxytocin as Anti-Obesity Treatment

### 8.1. Limitations in the Use of Oxytocin as an Anti-Obesity Medication and the Blood Brain Barrier

Oxt reduces body weight or weight gain in rodents and nonhuman primates, in part by reducing energy intake [90], and it reduces food intake following systemic, intranasal, or CNS administration [91]. However, in addition to a reduction in food intake, other mechanisms including energy expenditure also contribute to Oxt-elicited weight loss. Oxt stimulates BAT to help maintain body temperature, particularly during CS [2,44,45]. Oxt stimulates markers of thermogenesis in skeletal muscle, including uncoupling proteins. Oxt may help stimulate the transformation of white adipocytes to more metabolically active brown adipocytes [91]. These findings raise the possibility that Oxt may stimulate energy expenditure through multiple CNS and peripheral sites and raise the question as to the extent to which BAT thermogenesis and browning of WAT contribute to these effects. It will be important to determine if mice with global loss of Oxt/Oxtrs have impairment in both BAT thermogenesis and energy expenditure in response to cold exposure, and whether pretreatment with Oxt rescues both the impairment in BAT thermogenesis and energy expenditure. Oxt is important in muscle regeneration, and intranasal Oxt increases lean mass in senior men and women with sarcopenic obesity. Oxt does not cross the BBB, but some have raised the possibility that, with high peripheral doses, some Oxt is likely to enter the brain despite the presence of a very effective BBB to Oxt [92,93]. Similar to vasopressin, Oxt can stimulate its own release, which is why could be of pharmacological help to inhibit Oxt reuptake, similarly to a selective serotonin reuptake inhibitor (SSRI). Oxt may increase BAT thermogenesis through its action in the modulation of CNS sympathetic output and on sympathetic pre-ganglionic. Oxtr expressing neurons within the spinal cord to increase SNS outflow. Collectively, this may adversely affect cardiac function and hamper its translational potential [94]. While there is much enthusiasm over the potential use of Oxt as a therapeutic strategy to treat obesity, we need to wait the results of ongoing clinical trials in obese humans for additional confirmation of its feasibility as a long-term weight loss strategy and assessment of adverse side-effects.

### 8.2. Oxytocin in Clinical Trials

This excitement has translated to 535 completed, ongoing, or pending investigations in humans [93]. The barriers to the use of chronic treatment include concerns about Oxt downregulation of Oxtrs [95,96] and of course the effect on uterus that is estrogen-dependent. The use of Oxt could be optimal as an adjunct therapy for obesity rather than a monotherapy [97]. Thus, the combination of Oxt with other therapies that act in part to reduce food intake and increase energy expenditure may help to greater weight loss than either treatment alone [98]. Oxt treatment alone has been found to be effective in weight loss, appearing to be relatively modest after 4–8 weeks treatment period in diet-induced obese mice, rats, and humans compared to weight loss achieved after long-term treatment with combination therapies in humans [71,97]. Cagrililintide (amylin analogue) + semaglutide and Qsymia have resulted in a weight loss of approximately 17% and 10% of initial body weight, respectively [99,100]. The combined treatment of Oxt (fourth ventricular infusion) and the beta-3 receptor agonist CL-316243 was effective in eliciting greater weight loss compared to Oxt (7.8%) or CL-316243 (9.1%) alone [101]. These findings are consistent with other beta-3 receptor agonists and the FDA-approved beta-3 receptor agonist Mirabegron with respect to increasing energy expenditure [102,103]. For example, the effect of Oxt is effective in reducing hyperphagia and weight loss given in combination with opioid antagonist naltrexon [104]. This may result from the fact that opiate antagonists increase the release and potentiate the effects of Oxt [105]. Indeed, natrexone and Oxt act synergically to reduce intake of a high-fat high-sugar diet over a 24 day period in rats at doses that produce changes in the gene expression of several genes in the brain. Chronic Oxt treatment may prevent the drop in energy expenditure that occurs after prolonged reductions of food intake and weight loss [71]. However, there are limitations to the potential therapeutic use of Oxt linked to the pleiotropy and to the chronicity of the Oxt treatment where relatively high doses are usedm which may produce off-target effects such as adverse cardiovascular effects, anxiety, vomiting, and increased aggressiveness or a downregulation of Oxtr [106,107]. Lastly, an important point is to clarify the Oxt regulation of eating in females, as high doses of Oxt are required to reduce food intake in mice during the pro-estrous stage of the estrous cycle, an effect that is attributable to the estrogen surge at that time [108]; therefore, this area represents an important direction for future research.

## 9. Conclusions

**Figure 2:**
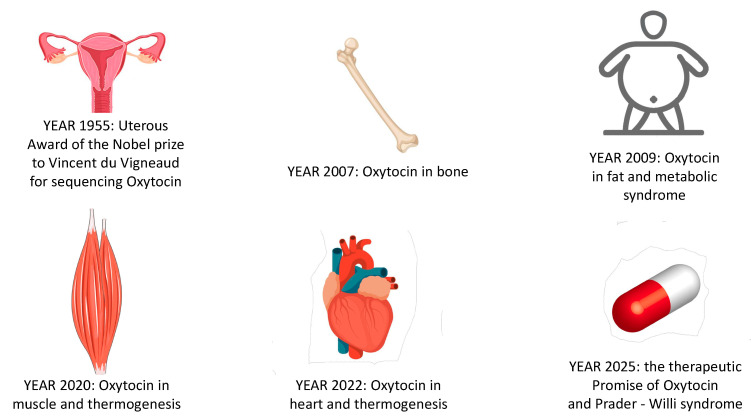
Seventy years of Oxytocin. This figure is a schematic representation of the groundbreaking discoveries of oxytocin’s functions over the past 70 years. In 1957, oxytocin was first sequenced, and its function as a uterotonic was established [8]. In 2007, oxytocin and oxytocin receptors were sequenced in osteoblasts, osteoclasts, and stem cells [22]. In 2009, oxytocin deficiency was unequivocally linked to fat and metabolic syndrome but in the absence of hyperphagia [35,36]. In 2016, oxytocin was found in muscle cells [2]. In 2020, oxytocin was shown to mediate thermoregulation and muscle contraction [3]. In 2022, a new pathway of oxytocin merging muscle, thermoregulation, myocardium functionality, and metabolism was discovered [4]. In 2025, it is estimated that oxytocin will be involved in Prader–Willi syndrome etiology, with anti-obesity properties [77,79].

Overall, Oxt has come a very long way since its first identification as a uterotonic, which was awarded with the Nobel Prize for chemistry in 1955 (Figure 2). However, the therapeutic use of Oxt as an anti-obesity medication still requires further studies, mostly addressing the difficulty of exogenous Oxt reaching the brain. Nevertheless, the physiology of Oxt can be useful to understand and treat diseases such as PWS and hypothalamic syndrome. We can set, as a new target, the award of another Nobel Prize for the discovery of Oxt’s therapeutic use in the treatment of PWS and obesity in humans.

## Data Availability

Data sharing not applicable to this article.

## Abbreviations

BBB	Blood–brain barrier
BAT	Brown adipose tissue
WAT	White adipose tissue
iWAT	Inguinal adipose tissue
CS	Cold stress
CSF	Cerebrospinal fluid
BBB	Blood brain barrier
HIPP	Hippocampus
MyHC	Myosin heavy chain
Oxt	Oxytocin
Oxtr	Oxytocin receptor
PVN	Paraventricular nucleus
PWS	Prader–Willi syndrome
SON	Supraoptical nucleus
Sol	Soleus muscle
TA	Tibialis anterioris
TRPV1	Transient receptor potential vanilloid 1
